# Immunoadsorption of Desmoglein-3-Specific IgG Abolishes the Blister-Inducing Capacity of Pemphigus Vulgaris IgG in Neonatal Mice

**DOI:** 10.3389/fimmu.2018.01935

**Published:** 2018-09-03

**Authors:** Maxi Hofrichter, Jenny Dworschak, Shirin Emtenani, Jana Langenhan, Fanny Weiß, Lars Komorowski, Detlef Zillikens, Winfried Stöcker, Christian Probst, Enno Schmidt, Stephanie Goletz

**Affiliations:** ^1^Lübeck Institute of Experimental Dermatology, University of Lübeck, Lübeck, Germany; ^2^Institute of Experimental Immunology, Euroimmun AG, Lübeck, Germany; ^3^Department of Dermatology, University of Lübeck, Lübeck, Germany

**Keywords:** acantholysis, autoantibody, desmoglein, desmosome, immunoadsorption, pemphigus, skin, treatment

## Abstract

Pemphigus vulgaris (PV) is a potentially life-threatening autoimmune blistering disease which is associated with autoantibodies directed against two desmosomal proteins, desmoglein (Dsg) 3 and 1. Treatment of PV is rather challenging and relies on the long-term use of systemic corticosteroids and additional immunosuppressants. More recently, autoantibody-depleting therapies such as rituximab, high-dose intravenous immunoglobulins, and immunoadsorption were shown to be valuable treatment options in PV. Specific removal of pathogenic autoantibodies would further increase efficacy and usability of immunoadsorption. Here, we tested the capacity of our recently developed prototypic Dsg1- and Dsg3-specific adsorbers to remove circulating pathogenic autoantibodies from three different PV patients. The pathogenic potential of the Dsg3/1-depleted IgG fractions and the anti-Dsg3-specific IgG was explored in two different *in vitro* assays based on cultured human keratinocytes, the desmosome degradation assay and the dispase-based dissociation assay. In addition, the neonatal mouse model of PV was used. In both *in vitro* assays, no difference between the pathogenic effect of total PV IgG and anti-Dsg3-specific IgG was seen, while Dsg3/1-depleted and control IgG were not pathogenic. For the samples of all 3 PV patients, depletion of anti-Dsg3/1 IgG resulted in a complete loss of pathogenicity when injected into neonatal mice. In contrast, injection of anti-Dsg3-specific IgG, eluted from the column, induced gross blistering in the mice. Our data clearly show that anti-Dsg3-specific IgG alone is pathogenic *in vitro* and *in vivo*, whereas Dsg3/1-depletion results in a complete loss of pathogenicity. Furthermore, our data suggest that Dsg-specific adsorption may be a suitable therapeutic modality to efficiently reduce pathogenic autoantibodies in patients with severe PV.

## Introduction

Pemphigus vulgaris (PV) is a potentially life-threatening intraepidermal blistering autoimmune disease (1–4). Desmoglein 3 (Dsg3) and desmoglein 1 (Dsg1) have been identified as autoantigens in PV (5–8). Dsg1 and Dsg3 are desmosomal transmembrane cadherins that mediate intercellular adhesion of keratinocytes in the skin and surface-close epithelia (3, 6, 9). In PV patients with exclusive mucosal involvement (mPV), autoantibodies are restricted to Dsg3, whereas autoantibodies against both Dsg3 and Dsg1 are associated with skin and mucosal lesions (mucocutaneous type of PV, mcPV) (10–12). In pemphigus foliaceus (PF), autoantibody reactivity is limited to Dsg1 and patients only develop skin lesions. In addition to Dsg1 and Dsg3, a variety of other target antigens have been described in PV including muscarinic and nicotinic acethylcholine receptors, annexins, thyroid peroxidase, desmocollins, and mitochondrial proteins (13–15). While good, albeit not undisputed, evidence for the pathogenic effect of anti-Dsg1/3 antibodies has been provided, less data were reported about the pathogenicity of non-Dsg antibodies (13, 15–20).

Treatment of PV is challenging and has required the long-term use of prednisolone and other immunosuppressants such as azathioprine and mycophenoles (4, 21, 22). Very recently, first-line rituximab, an anti-CD20 antibody that depletes B cells from the circulation for 3–9 months, in conjunction with the short term use of prednisolone has been shown to be significantly more effective and safe compared to the long term use of prednisolone alone (23). High-dose intravenous immunoglobulin and immunoadsorption are two other treatment modalities that reduce serum anti-Dsg autoantibodies and are recommended in refractory and/or severely affected PV patients (21, 22, 24). The reduction of serum autoantibodies in PV appears to be a particularly attractive therapeutic approach since the direct pathogenic importance of pemphigus autoantibodies has been shown in various experimental settings *in vitro* and *in vivo* (13, 25).

Whereas plasmapheresis requires substitution with fresh-frozen plasma or human albumin, immunoadsorption specifically removes antibodies from the circulation (24). Unfortunately, the use of immunoadsorption is limited by the increased risk of infections due to the parallel reduction of protective immunoglobulins. Thus, removal of Dsg-specific antibodies appeared to be advantageous leading to the recent development of prototypic anti-Dsg1 and anti-Dsg3 adsorbers. The Dsg1/3-specific adsorbers are based on the recombinant Dsg ectodomains coupled to sepharose and allowed the effective removal of anti-Dsg reactivity from PV and PF serum samples *in vitro* (26). The aim of the present study was to show that by the use of the Dsg1/3-specific adsorbers removal of anti-Dsg antibodies from PV sera is sufficient to abolish the pathogenic effect of pemphigus IgG not only *in vitro* but also *in vivo* in neonatal mice. We now also show that anti-Dsg3-specific IgG is sufficient for acantholysis in cultured keratinocytes and blister formation in neonatal mice.

## Material and methods

### Patients

IgG from 3 PV patients (PV1, PV2, PV3) that were treated with conventional protein A immunoadsorption at the Department of Dermatology, Lübeck, was used (27, 28). The clinical phenotype, age, sex, indirect immunofluorescence (IF) serum titers on monkey esophagus, and anti-Dsg1/3 IgG serum levels by ELISA (Euroimmun, Lübeck, Germany) are shown in Table 1. IgG bound to protein A was eluted by glycine buffer (pH 2.8) and immediately neutralized with 1M Tris pH 9.0 followed by precipitation with ammonium sulfate and dialysis against PBS. As no immunoadsorption material from healthy donors is available, we use affinity-purified IgG from sera of healthy volunteers as control. The study was performed following the Declaration of Helsinki. Pathogenicity experiments were positively reviewed by the ethics committees of the University of Lübeck, Germany (file reference, 09-090).

**Table 1:** Pemphigus vulgaris (PV) patients' characteristics.

**Patient no**.	**Sex**	**Age (years)**	**Clinical phenotype**	**Monkey esophagus (serum)**	**Anti-Dsg1 serum level (U/ml)**	**Anti-Dsg3 serum level (U/ml)**
PV1	M	70	mcPV	1:2,560	1,045	3,572
PV2	M	44	mcPV	1:1,280	103	9,748
PV3	F	72	mPV	1:320	–	6,001

*mcPV, mucocutaneous PV; mPV, mucosal PV*.

### Affinity purification of Dsg-specific PV IgG using the entire ectodomain of Dsg3 Dsg1

For antigen-specific immunoaffinity purification of anti-Dsg3 and anti-Dsg1 IgG, the entire ectodomains of Dsg3 and Dsg1, respectively, were immobilized on N-hydroxysuccinimide-activated Sepharose 4 Fast Flow (GE Healthcare, Buckinghamshire, UK) as previously described (26). Immunoaffinity purifications were performed as follows. The immobilized protein matrix was transferred into microcentrifuge spin columns (Thermo Fisher Scientific, Darmstadt, Germany) and washed three times with tris-buffered saline supplemented with 5 mM CaCl_2_ (Ca^2+^-TBS). The concentrated IgG of the PV patients was diluted 1:1 with Ca^2+^-TBS and incubated with the immobilized protein for 30 min at room temperature. The flow-through fraction was collected by centrifugation at 500x g for 30 s. After several washing steps with Ca^2+^-TBS (until OD_280_ < 0.05) the anti-Dsg3 and anti-Dsg1 IgG fractions were eluted from the matrix with IgG elution buffer (Thermo Fisher Scientific) until the OD_280_ was below 0.05, and immediately neutralized with 1M Tris pH 9.0. All eluted fractions were pooled and buffer was exchanged to PBS using Vivaspin 500 centrifugal filter units (Sartorius AG, Göttingen, Germany). Finally, Dsg3/1-depleted PV IgG (flow-through fractions) and anti-Dsg3 PV IgG (eluted fractions) were analyzed for anti-Dsg3 and anti-Dsg1 autoantibody reactivity by ELISA (Euroimmun).

### Immunoblotting with HaCaT extract

HaCaT cells were grown in low calcium Keratinocyte Growth Medium 2, KGM2 (Promocell, Heidelberg, Germany) containing 0.06 mM CaCl2 to confluence and lysed in Laemmli sample buffer. Lysates were fractionated by SDS-PAGE, transferred to nitrocellulose membrane and immunoblotted as reported (29). After blocking, nitrocellulose membranes were incubated with anti-Dsg3 specific IgG (2 μg/ml), a monoclonal anti-Dsg3 antibody (1:100, Bio-Rad, Munich, Germany), control IgG (2 μg/ml) from a healthy donor and IVIg (2 μg/ml, Biotest, Dreieich, Germany) diluted in TBST containing 5% skimmed milk powder plus 1% BSA. As secondary antibodies a horseradish peroxidase (HRP)-conjugated polyclonal goat anti-human IgG antibody (1:1,000, DAKO, Hamburg, Germany,) and a polyclonal rabbit anti-mouse IgG antibody (1:1,000; DAKO) were used. The proteins were visualized using Super Signal West Femto (Thermo Fisher Scientific).

### Desmosome degradation assay

The desmosome degradation assay was performed as described previously (26, 30, 31). In brief, HaCaT cells were grown in 8-well chamber slides (BDBiosciences, Heidelberg, Germany) to confluent monolayers. Low calcium Keratinocyte Growth Medium 2, KGM2 (Promocell) containing 0.06 mM CaCl_2_ was changed to high calcium medium by adding sterile 0.15 M CaCl_2_ to a final concentration of 1.5 mM calcium. Monolayers were treated with PV IgG, control IgG and IgG fractions collected from Dsg3 and Dsg1 immunoaffinity purification as indicated in the results part. After 24 h incubation at 37°C in a humidified atmosphere, culture medium was removed and monolayers were washed with DPBS (Thermo Fisher Scientific) and subsequently fixed with 4% paraformaldehyde. After washing, monolayers were treated with 0.1% Triton X-100 (Sigma Aldrich, Steinheim, Germany), incubated with 10% normal goat serum (DAKO) plus 1% BSA (Carl Roth, Karlsruhe, Germany) and then with mouse anti-human Dsg3 IgG1 (1:100 in PBS, clone 5G11; Acris, Herford, Germany) for 30 min at 37°C and after three times washing with Cy3-labeled goat anti-mouse IgG (1:100 in PBS; Dianova, Hamburg, Germany). Slides were mounted with ProLong R Gold antifade reagent (Life Technologies, Carlsbad, USA) and examined microscopically (BZ-9000, Keyence, Neu-Isenburg, Germany).

### Dispase-based dissociation assay

The assay was performed as reported previously (26, 30–32) with minor modifications. In brief, HaCaT cells were cultivated in 12-well-plates (Greiner Bio-One, Solingen, Germany) with KGM2 (Promocell) containing 0.06 mM CaCl_2_ in a humidified atmosphere (5 CO_2_) at 37°C. At confluence, fresh KGM2 containing 1.5 mM CaCl_2_ was added. PV IgG (2 mg/mL), control IgG (2 mg/mL), anti-Dsg3-specific IgG fractions collected from Dsg3- and Dsg1-specific affinity purification (20 μg/mL) and anti-Dsg3/1-depleted IgG (2 mg/mL), respectively were added and incubated for 24 h. Subsequently, the cells were treated with dispase solution (2.5 U/ml for 30 min; Stemcell Techn., Vancouver, Canada) and subjected to mechanical stress by pipetting 10 times (5 times moderate, 5 times strong) with a 1 ml pipette. Cell fragments were fixed and stained with crystal violet (Sigma Aldrich). Photos were taken from each well and cell fragments were counted manually. Every experiment was performed at least in triplicate.

### Passive transfer neonatal mouse model

Neonatals from C57BL/6 mice (Charles River Laboratories, Sulzfeld, Germany) were injected subcutaneously <24 h after birth with the respective PV IgG fraction at doses of 3–7 mg/g of total and anti-Dsg3/1-depleted IgG and 300 μg/g anti-Dsg3-specific IgG (each IgG batch was applied in 3 mice) with or without exfoliative toxin A (ETA; Toxin Technology Inc., Sarasota, USA) as described in parts previously (33, 34). ETA is a serine protease produced from *Staphylococcus aureus* which specifically degrades Dsg1 (35). Due to the different expression patterns of Dsg1 and Dsg3 in mucous membranes and the skin, anti-Dsg3 IgG is only pathogenic in the skin when Dsg1 is graded concomitantly (either by anti-Dsg1 IgG or ETA). In contrast, in mucous membranes, anti-Dsg3 antibodies alone are sufficient to induce intraepithelial splitting (12). Here, half of the minimum ETA dose was applied that in preliminary experiments had induced clinical blistering (usually 0.1 μg/g bodyweight). After 16–24 h, the mice were clinically evaluated before and after application of mechanical stress at the back and sides of mice (Nikolsky phenomenon). Blood was obtained as well as biopsies from the back for histopathology (H&E staining) and direct IF microscopy. All animal experiments were approved by the Animal Rights Commission of the Ministry of Agriculture and Environment, Schleswig Holstein (98-8/14).

### Immunofluorescence microscopy

For direct IF microscopy, a polyclonal rabbit anti-human IgG-FITC antibody (Bio-Rad, Hercules, USA) at a dilution of 1:50 in PBS was used for 1 h at room temperature. For indirect IF microscopy, 6 μm sections of monkey esophagus were incubated with human IgG and mouse sera in a dilution range of 1:20–1:5120 in PBS for 1 h at room temperature. For detection, a FITC-labeled polyclonal anti-human IgG (DAKO) at 1:50 in PBS was employed for 30 min at room temperature.

### Statistics

Graphpad Prism 6 was used for the statistical analysis. The dispase assay data across different groups within each patient was compared for its statistical significance using the Kruskal-Wallis test. For all three patients, correction for multiple comparisons was done by *post-hoc* Dunn's tests to identify significant pairwise differences between the groups.

## Results

### Dsg1- and Dsg3-specific adsorption of PV patient IgG

**Figure 1:**
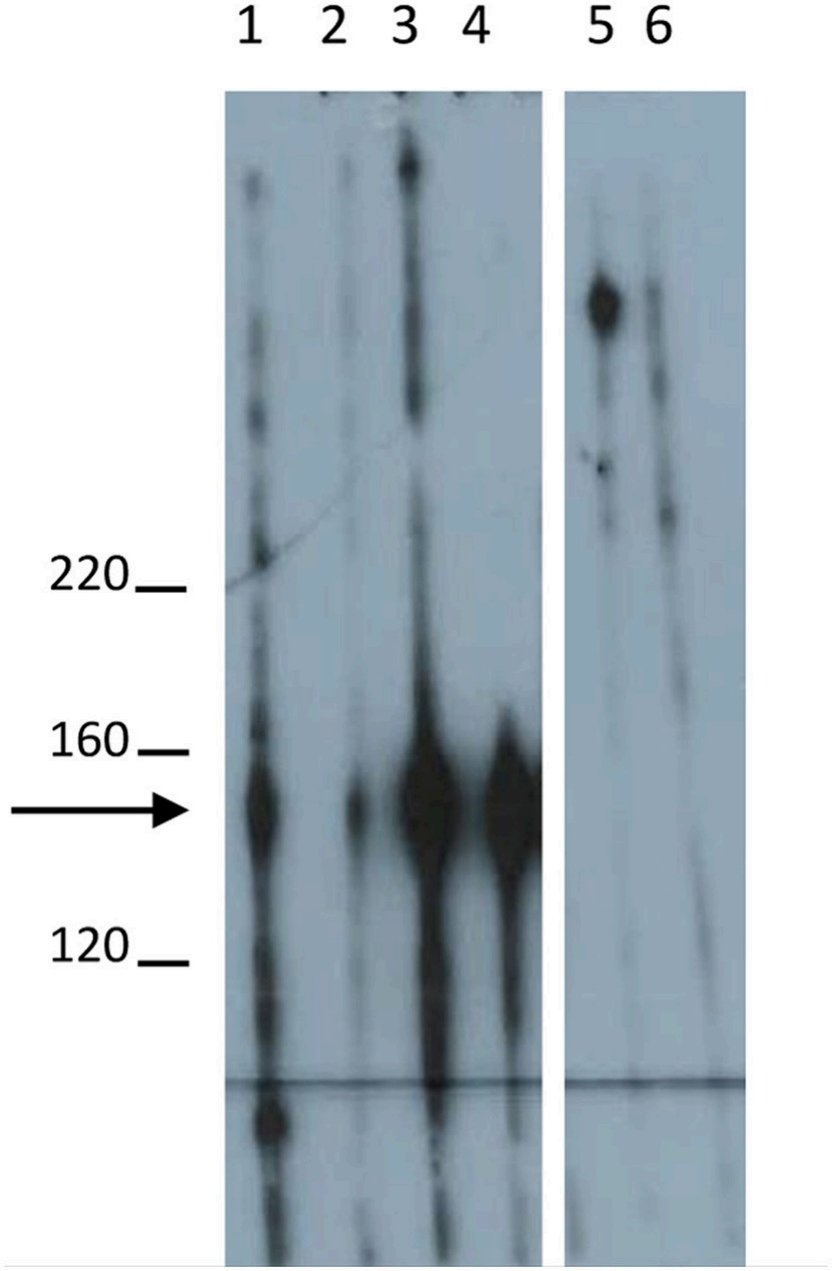
Desmoglein3-specific IgG reactivity in Western blot using extract of cultured keratinocytes. Western blotting of a monoclonal anti-Dsg3 antibody (lane 1; Bio-Rad, Munich, Germany), Dsg3-specific IgG (2 μg/ml) from pemphigus vulgaris (PV) patient PV1 (lane 2), PV2 (lane 3), and PV3 (lane 4) with extract of cultured HaCaT keratinocytes. IgG affinity purified from a healthy volunteer (2 μg/ml) and IVIg (2 μg/ml, Biotest, Dreieich, Germany) are shown in lanes 5 and 6. Molecular weight markers are shown to the left (kDa). The arrow indicates the migration position of desmoglein 3.

**Figure 2:**
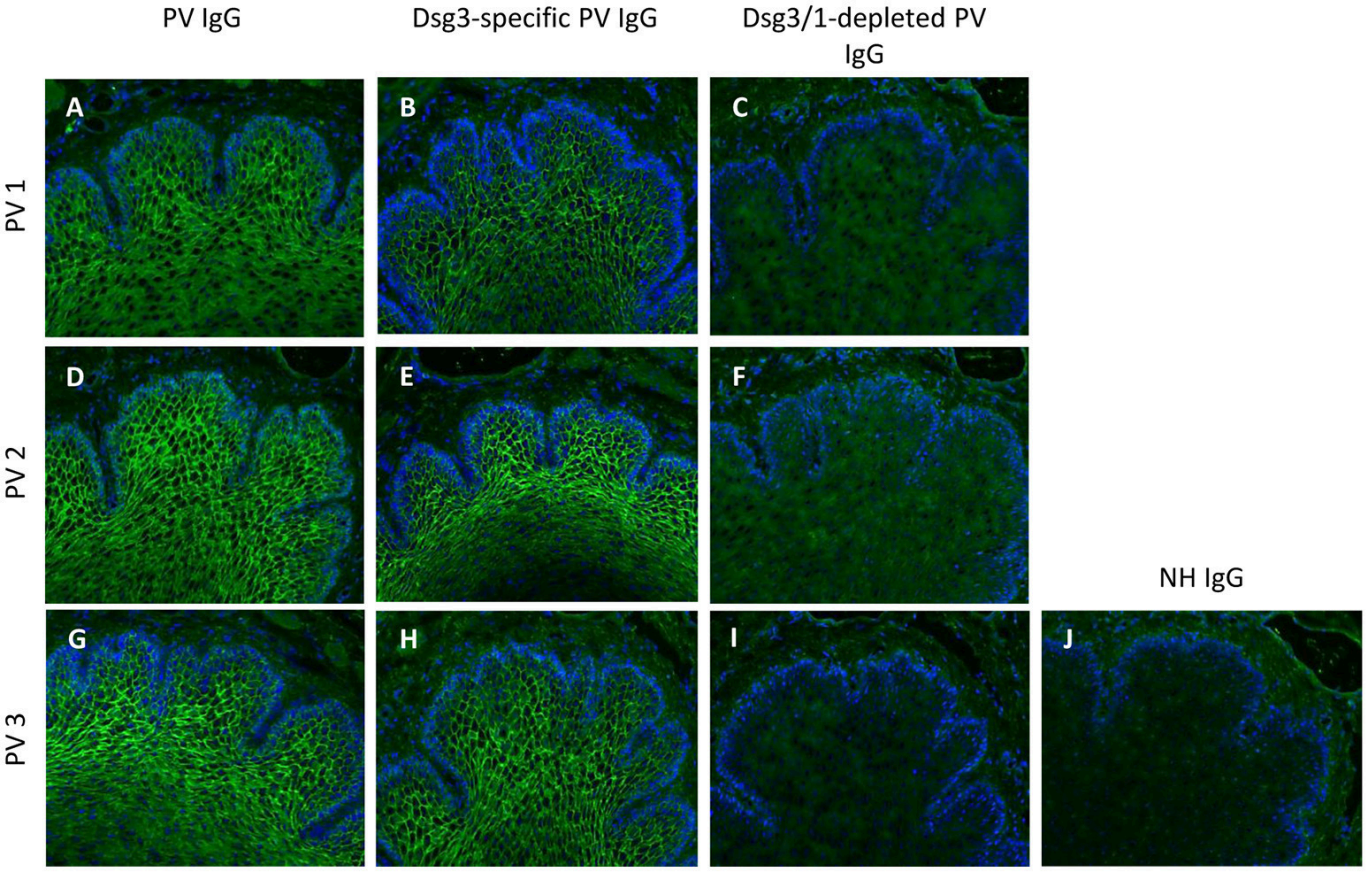
Indirect immunofluorescence (IF) microscopy on monkey esophagus confirms Dsg3/1-specific depletion of IgG from pemphigus vulgaris (PV) IgG. Total PV IgG from three different pemphigus vulgaris (PV) patients (PV1 **(A)**, PV2 **(D)**, PV3 **(G)**) and the respective anti-Dsg3-specific IgG **(B,E,H)** but not the anti-Dsg3/1 IgG-depleted IgG fractions **(C,F,I)** and normal human IgG (NH IgG; **J**) revealed the characteristic intercellular epithelial staining. Nuclei were counterstained with DAPI.

Anti-Dsg3-specific IgG was immunoadsorbed from total PV IgG in all three PV patients as previously described in Langenhan et al. (26). In addition, in PV1 and PV2, the present Dsg1-reactivity was removed by Dsg1-specific immunoadsorption. To study the effect of Dsg-specific IgG we focused on anti-Dsg3-specific IgG since only two of the three PV patients revealed Dsg1-specifc IgG. Characteristics of patient IgG after Dsg3/1-specific adsorption are summarized in Tables 2, 3. Western blot analysis of cellular extracts of cultured human keratinocytes confirmed the specific purification of anti-Dsg3 PV IgG (Figure 1). Indirect IF microscopy on monkey esophagus revealed that total IgG from PV patients as well as Dsg3-specific IgG, but not Dsg3/1-depleted IgG and control IgG from a healthy blood donor, showed the PV-typical intercellular staining of the stratified squamous epithelium (Figure 2, Table 2).

**Table 2:** Characteristics of pemphigus vulgaris (PV) IgG fractions.

**Patient no**.	**PV IgG^a^**			**Purified anti-Dsg3 PV IgG**			**Anti-Dsg3/1 depleted PV IgG**		
	**Dsg3 (U/ml)^b^**	**Dsg1 (U/ml)^b^**	**IIF (titer)^c^**	**Dsg3 (U/ml)^b^**	**Dsg1 (U/ml)^b^**	**IIF (titer)^c^**	**Dsg3 (U/ml)^b^**	**Dsg1 (U/ml)^b^**	**IIF (titer)^c^**
PV1	49,446	16,933	>1:5,120	9,463	Neg.	1:640	Neg.	Neg.	Neg.
PV2	29,526	27	>1:5,120	19,034	Neg.	1:1,280	Neg.	Neg.	Neg.
PV3	11,773	Neg.	>1:5,120	1,691	Neg.	1:320	Neg.	Neg.	Neg.

a *Before subjection to Dsg-specific adsorption*.

b *By ELISA (Euroimmun; lower cut-off 20 U/ml)*.

c *By indirect immunofluorescence (IIF) microscopy on monkey esophagus. Dsg, desmoglein; neg., negative*.

**Table 3:** Characteristics of pemphigus vulgaris (PV) IgG fractions (dispase-based dissociation assay).

**Patient no**.	**PV IgG^a^**		**Purified anti-Dsg3 PV IgG**		**Anti-Dsg3/1 depleted PV IgG**	
	**Dsg3 (U/ml)^b^**	**Dsg1 (U/ml)^b^**	**Dsg3 (U/ml)^b^**	**Dsg1 (U/ml)^b^**	**Dsg3 (U/ml)^b^**	**Dsg1 (U/ml)^b^**
PV1	1,776	44	139	Neg.	Neg.	Neg.
PV2	3,217	Neg.	1,446	Neg.	Neg.	Neg.
PV3	2,274	Neg.	752	Neg.	Neg.	Neg.

a *Before subjection to Dsg-specific adsorption*.

b By ELISA (Euroimmun; lower cut-off 20 U/ml).

*Dsg, desmoglein; neg., negative*.

### Dsg3/1-specific depletion of PV IgG abolishes pathogenicity *in vitro*

**Figure 3:**
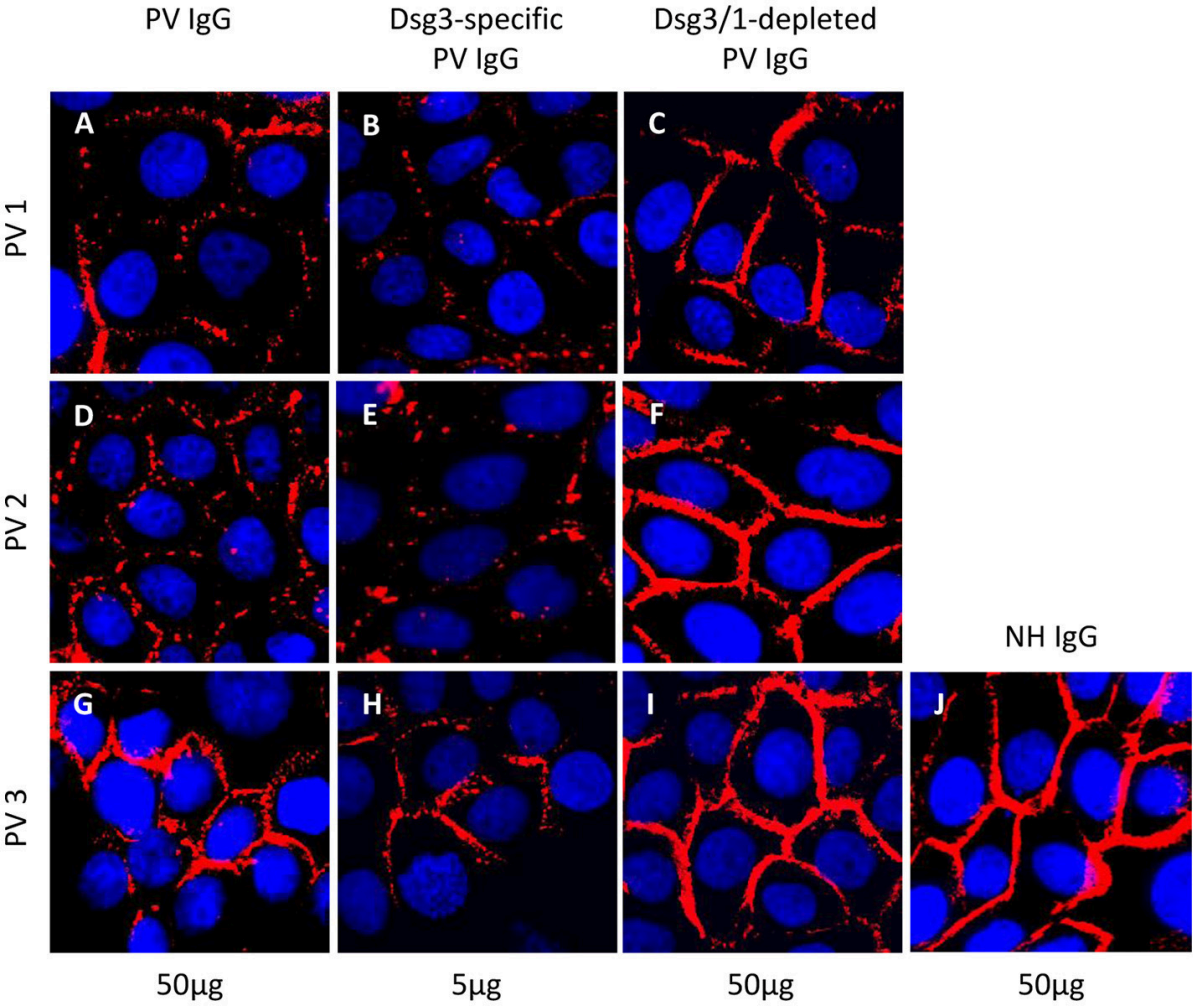
Desmosome degradation assay. HaCaT keratinocytes were treated with 50 μg/ml total pemphigus vulgaris (PV) IgG, 5 μg/ml anti-Dsg3-specific and 50 μg/ml anti-Dsg3/1 IgG-depleted PV IgG from three different pemphigus vulgaris (PV) patients (PV1, PV2, PV3) before immunostaining with anti-Dsg3 IgG. Dsg3 degradation was detected after incubation with total PV IgG **(A,D,G)** and Dsg3-specific IgG **(B,E,H)** but not with the PV IgG fractions depleted of anti-Dsg1/3 IgG **(C,F,I)** and normal human IgG (50 μg/ml; NH IgG; **J**).

To evaluate the pathogenicity of the Dsg3-specific PV IgG and anti-Dsg3/1 IgG-depleted fractions *in vitro*, the desmosome degradation assay and the dispase-based dissociation assay were performed. The PV IgG-induced loss of Dsg3 expression on the keratinocyte cell surface due to internalization of Dsg3 into endosomes and degradation was determined microscopically. Incubation of HaCaT cell monolayers with either total PV patient IgG or anti-Dsg3-specific IgG resulted in an equivalent discontinuous Dsg3 staining at the keratinocyte cell borders (Figure 3). In contrast, when cells were treated with anti-Dsg3/1 IgG-depleted fractions and normal human IgG, respectively, Dsg3 staining was uniformly localized to the cell membrane of keratinocytes (Figure 3).

**Figure 4:**
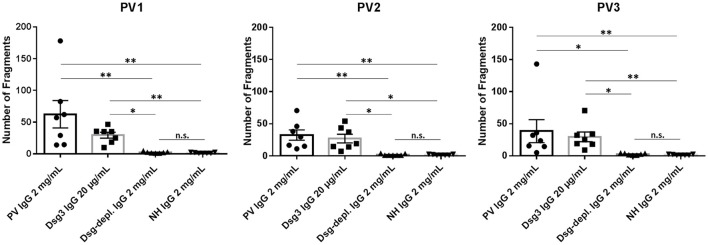
Dispase-based dissociation assay. Incubation of keratinocyte monolayers with total pemphigus vulgaris (PV) IgG and anti-Dsg3-specific IgG from three different PV patients (PV1, PV2, PV3) showed a significantly higher fragmentation compared to treatment with normal human IgG (NH IgG) and the anti-Dsg3/1-depleted PV IgG (Dsg-depl. IgG), respectively. No difference between incubation with NH IgG and anti-Dsg3/1-depleted IgG was observed. In addition, incubation with Dsg3-specific IgG resulted in significantly higher fragmentations compared to both Dsg-depleted PV IgG and NH IgG, respectively. Data show the mean and standard error of the mean (error bars) of seven independent experiments. *, *p* < 0.05; **, *p* < 0.01; n.s., not significant.

In the dispase-based dissociation assay, treatment with PV1, PV2, and PV3 IgG, respectively, as expected resulted in significantly more keratinocyte fragments compared to incubation with normal human IgG (PV1: *p* = 0.0011; PV2: *p* = 0.0045; PV3: *p* = 0.003; Figure 4). Incubation of monolayers with purified Dsg3-specific PV IgG from the three patients generated a significantly higher fragmentation level compared to incubation with anti-Dsg3/1-depleted IgG (PV1: *p* = 0.046; PV2: *p* = 0.011; PV3: *p* = 0.02; Figure 4). No difference was observed between treatment with anti-Dsg3/1-depleted IgG and normal human IgG (Figure 4).

### Anti-Dsg3/1 IgG-depleted PV IgG prevents pathogenicity while anti-Dsg3-specific IgG results in blister formation in neonatal mice

**Figure 5:**
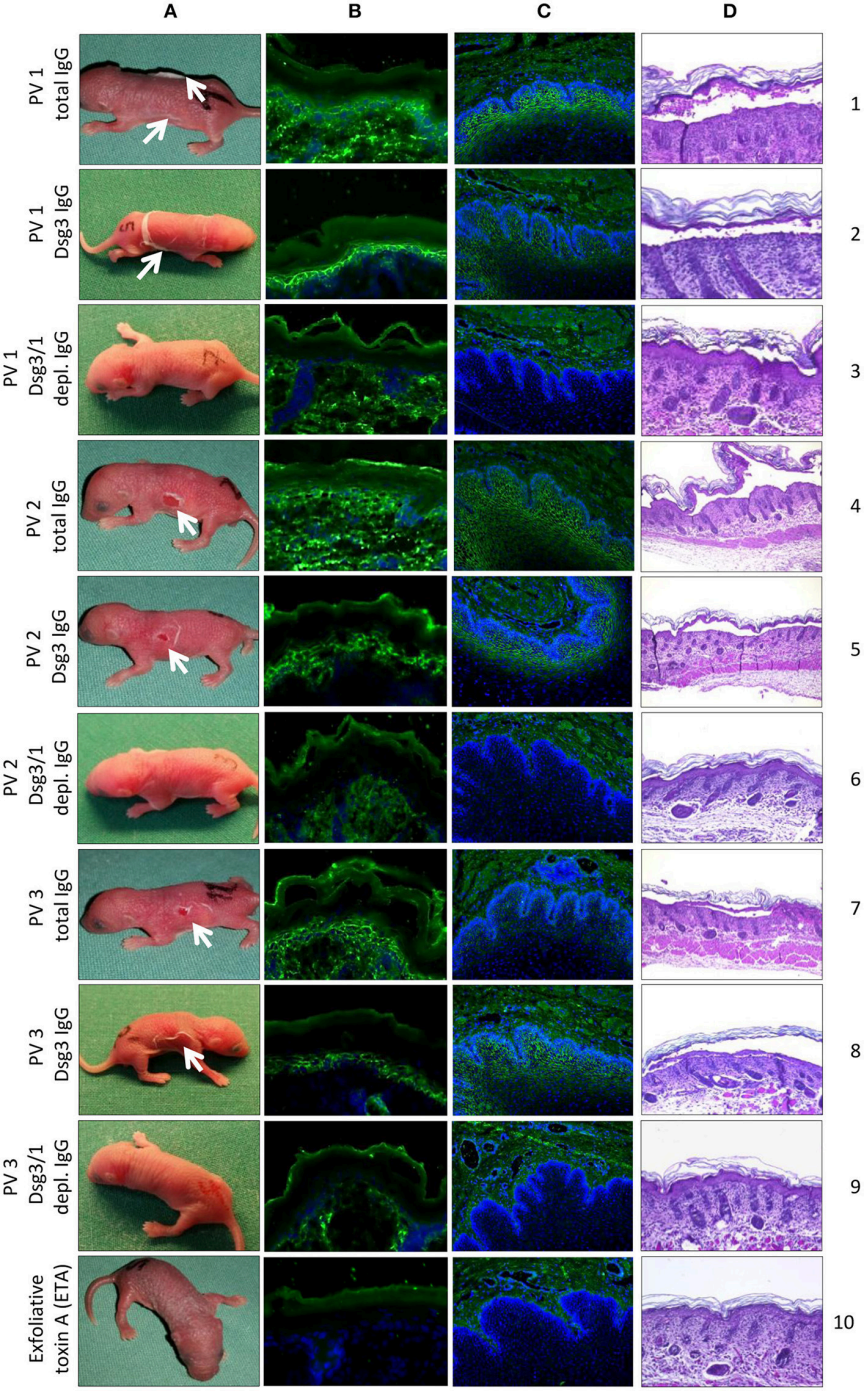
*In vivo* pathogenicity of pemphigus vulgaris (PV) IgG fractions. Injection of neonatal mice (*n* = 3/ group) with PV IgG and anti-Dsg3-specific IgG purified from three different PV patients PV1, PV2, PV3) induced flaccid macroscopic blisters (**A**; lanes 1, 2, 4, 5, 7, and 8; white arrows) and suprabasal splitting as seen by lesional histopathology (**D**; lanes 1, 2, 4, 5, 7, and 8). No macroscopic and microscopic blistering was induced by PV IgG depleted of anti-Dsg3/1 IgG from the three PV patients (**A,D**; lanes 3, 6, and 9) or ETA alone (**A,D**; lane 10). By direct immunofluorescence (IF) microscopy of back skin, an intercellular epidermal staining was observed in mice injected with PV IgG (**B**; lanes 1, 4, and 7) or anti-Dsg3-specific IgG (**B**; lanes 2, 5, and 8) but not after injection of anti-Dsg3/1 IgG-depleted IgG (**B**; lanes 3, 6, and 9) or ETA alone (**B**, lane 10). By indirect IF microscopy on monkey esophagus, the characteristic intercellular staining (1:80 dilutions are shown) was seen with sera of mice injected with PV IgG (**C**; lanes 1, 4, and 7) and anti-Dsg3-specific PV IgG (**C**; lanes 2, 5, and 8), but not after injection of PV IgG depleted of Dsg3-specific IgG (**C**; lanes 3, 6, and 9) or ETA alone (**C**, lane 10). Nuclei were counterstained with DAPI.

When injected into neonatal mice, only total PV1 IgG contained enough anti-Dsg1 antibodies for the induction of skin blisters without co-injection of a subclinical dose of ETA (Figure 5A, lane 1). For mice injected with PV2 or PV3 IgG, gentle mechanical friction was required to obtain macroscopic blistering (Figure 5A, lanes 4 and 7). The injection of anti-Dsg3-specific IgG fractions from all three PV patients (combined with subclinical ETA doses) induced gross skin blistering (Figure 5A, lanes 2, 5, and 8). In contrast, anti-Dsg3/1 IgG-depleted IgG from all three PV patients (combined with subclinical ETA doses) failed to induce blistering in neonatal mice (Figure 5A, lanes 3, 6, and 9). Lesional skin biopsies revealed suprabasal acantholysis, the characteristic histological finding of PV, after injection of PV IgG and anti-Dsg3-specific IgG, but not after injection of Dsg3/1-depleted PV IgG fractions or ETA alone (Figure 5D). By direct IF microscopy, intercellular IgG depositions were found in the epidermis in all PV IgG and anti-Dsg3-specific IgG-injected mice, but not in mice injected with anti-Dsg3/1 IgG-depleted PV IgG or ETA alone (Figure 5B). By indirect IF microscopy on monkey esophagus, the characteristic intercellular staining was seen with sera of mice injected with PV IgG and anti-Dsg3 specific PV IgG, but not after injection of anti-Dsg1/3-depleted IgG or ETA alone (Figure 5C).

## Discussion

Adjuvant immunoadsorption is a well-established treatment option in a variety of autoantibody-mediated diseases including PV. So far, more than 100 pemphigus patients were reported to have been subjected to immunoadsorption which has been recommended in the guideline of the German Dermatological Society for the treatment of refractory or severe PV (21, 36). Furthermore, the results of a randomized control trial comparing the efficacy and safety of immunoadsorption plus best medical treatment with best medical treatment alone in pemphigus are currently evaluated. In PV, it may be of particular value to rapidly reduce the amount of circulating autoantibodies at the beginning of treatment at a stage when other therapies, i.e., corticosteroids, azathioprine, and rituximab, are not yet effective. This assumption is supported by the clear evidence of a direct pathogenic effect of pemphigus autoantibodies as demonstrated by the occurrence of transient pemphigus in neonates of mothers with PV, the correlation of disease activity with serum levels of anti-Dsg1/3 IgG and various experimental models *in vitro* and *in vivo* (2, 15, 20, 33). Conventional immunoadsorption is, however, limited due to the risk of hypogammaglobulinaemia and the subsequent risk of infections. This disadvantage would not be applicable for the use of autoantibody-specific adsorbers. Therefore, we have recently developed Dsg1- and Dsg3-specific adsorbers based on the recombinant Dsg ectodomains. We could show that the prototypic adsorbers effectively removed anti-Dsg1/3 IgG from PV and PF sera and eliminated the pathogenic effect of PV and PF IgG *in vitro* (26).

In the present study, the prototypic adsorbers were employed to investigate whether Dsg1/3-specific adsorption can also abolish the pathogenic effect of PV IgG *in vivo*. Extending our previous studies (26), we asked the question whether anti-Dsg3-specific IgG alone, i.e., without the addition of non-desmoglein antibodies, is sufficient to induce pathogenic effects *in vitro* and intraepidermal blistering in mice. Our experiments are of particular importance since the concept that in the great majority of PV patients, the pathogenic effects of autoantibodies are mediated by anti-Dsg antibodies is challenged (13, 16). Furthermore, although Amagai et al. previously showed that affinity-purified anti-Dsg3 IgG prevented pathogenicity *in vivo* (37), there are still doubts about the specificity of the affinity purification as the recombinant Dsg3 fragment used for this process contained the constant region of human IgG1 that might have also bound to non-Dsg PV autoantibodies (13, 38). In our Dsg1/3-specific adsorbers, only the ectodomains of Dsg1 and 3 were used (26).

Here, we initially demonstrated the high efficiency of the Dsg3/1-specific adsorbers since no anti-Dsg3 or anti-Dsg1 IgG antibodies could be detected in the Dsg3/1-depleted IgG fraction by ELISA. This result was corroborated by Western blotting of anti-Dsg1/3-specific IgG and anti-Dsg1/3 IgG-depleted PV IgG fractions with extract of human keratinocytes. In line, by indirect IF microscopy on monkey esophagus both, total PV IgG and anti-Dsg3-specific IgG but not anti-Dsg3/1 IgG-depleted PV IgG and normal human IgG stained the epithelium.

Next, we demonstrated in two different *in vitro* assays that PV IgG, depleted from anti-Dsg1/3 reactivity by the use of the Dsg1/3-specific adsorbers, lost their pathogenic effect. No difference between anti-Dsg1/3 IgG-depleted IgG and normal human IgG was observed in both, the desmosome degradation assay and the dispase-based dissociation assay. In contrast, PV IgG and anti-Dsg3-specific IgG obtained after elution from our Dsg1/3-specific adsorbers led to increased desmosome degradation and keratinocyte dissociation, respectively. It has already previously been shown that human keratinocytes loose Dsg3 expression on their cell surface after incubation with PV IgG (39, 40). Nevertheless, we observed that total PV IgG and anti-Dsg3-specific IgG from PV3 resulted in less Dsg3 degradation (Figures 3G,H) compared to the IgG fractions of PV1 and PV2. We hypothesize that the weaker desmosome-degrading capacity of both PV3 IgG and PV3 anti-Dsg3-specific IgG may be explained by the lower anti-Dsg3 IgG titers in this patient (Table 2).

Furthermore, the different PV IgG fractions were also assayed in the neonatal mouse model of PV. Initially, Anhalt and coworkers reported that the injection of PV serum in neonatal mice recapitulated major clinical and immunopathological characteristics of the human diseases, i.e., flaccid blisters that easily erode when mechanical friction is applied, intraepidermal split formation as detected by histopathology, and the intercellular binding of PV antibodies in the epidermis as seen by direct IF microscopy (33). In the present study, the injection of PV IgG and Dsg1/3-specific IgG led to macroscopic and microscopic blisters indicating that anti-Dsg1/3 IgG alone is pathogenic and does not require the presence of non-Dsg PV autoantibodies. These data are supported by the previous observations that injection of the monoclonal anti-Dsg3 antibody AK23 resulted in blister formation in neonatal as well as in adult mice (41, 42). More important for the future use of the Dsg1/3-specific adsorbers in the treatment of PV patients is our observation that PV IgG fractions depleted from anti-Dsg1/3 reactivity did not induce skin lesions when injected into neonatal mice. These results unequivocally show that non-Dsg antibodies that had been previously described in PV sera directed e.g., against muscarinic and nicotinic acethylcholine receptors, annexins, thyroid peroxidase, and mitochondrial proteins are not a prerequisite for blister formation in PV. In line, these non-Dsg antibodies have not yet been described to be pathogenic *in vivo* while co-pathogenic effects have been reported *in vitro* (13, 15, 17, 18, 20, 43, 44). One may speculate that the previously proposed pathogenic effect of non-Dsg antibodies in PV is not a key element for the initiation of blister formation.

In contrast, anti-desmocollin autoantibodies that have been described in pemphigus sera caused desmosome degradation in the desmosome degradation assay, cell fragmentation in the dispase-based dissociation assay, and suprabasal splitting in an *ex vivo* skin model (45–47). In line, desmocollin 3-deficient mice present with skin erosions and suprabasal intraepidermal blistering (48). However, evidence is accumulating that anti-desmocollin autoantibodies may be more relevant in paraneoplastic and atypical pemphigus than in PV and PF (49–51). In fact, in a large prospective study with more than 330 pemphigus patients, only 4% of all pemphigus sera and 2.7% of PV and PF sera exhibited anti-desmocollin reactivity, while 98% of sera contained anti-Dsg3 and/or anti-Dsg1 IgG (52). These data indicate that only in a small number of PV and PF patients, Dsg1/3-specific immunoadsorption may not be clinically effective although anti-Dsg1/3 antibodies have effectively been decreased. Future studies now aim at applying the Dsg3/1-specific adsorbers in a clinical trial with PV patients.

## Author contributions

MH contributed to the performance of the experiments and the writing of the manuscript. JD contributed to the planning of the project and the performance of the experiments. SE and JL contributed to the performance of the experiments and to the revision of the manuscript. FW contributed to the performance of the experiments and to the revision of the manuscript. LK contributed to the planning of the project. DZ contributed to the planning of the project and to the revision of the manuscript. WS contributed to the planning of the project and to the revision of the manuscript. CP contributed to the planning of the project and to the revision of the manuscript. ES contributed to the planning of the project and to the writing of the manuscript. SG contributed to the planning of the project and to the writing of the manuscript.

### Conflict of interest statement

JD, JL, LK, and CP are employees of Euroimmun AG. Winfried Stöcker is board members of Euroimmun AG. DZ and ES have a research cooperation with Euroimmun. The remaining authors declare that the research was conducted in the absence of any commercial or financial relationships that could be construed as a potential conflict of interest.

## Abbreviations

Dsg: desmoglein
IF: immunofluorescence
NH IgG: normal human immunoglobulin G
PF: pemphigus foliaceus
PV: pemphigus vulgaris
mPV: mucosal pemphigus vulgaris
mcPV: mucocutaneous pemphigus vulgaris.

## References

[1] Schmidt E Zillikens D. The diagnosis and treatment of autoimmune blistering skin diseases. Dtsch Arztebl Int. (2011) 108:399–405. 10.3238/arztebl.2011.0405 21712975 PMC3123771

[2] Kasperkiewicz M Ellebrecht CT Takahashi H Yamagami J Zillikens D Payne AS. Pemphigus. Nat Rev Dis Primers. (2017) 3:17026. 10.1038/nrdp.2017.26 28492232 PMC5901732

[3] Ludwig RJ Vanhoorelbeke K Leypoldt F Kaya Z Bieber K McLachlan SM. Mechanisms of autoantibody-induced pathology. Front Immunol. (2017) 8:603. 10.3389/fimmu.2017.00603 28620373 PMC5449453

[4] Schmidt E. Rituximab as first-line treatment of pemphigus. Lancet (2017) 389:1956–8. 10.1016/S0140-6736(17)30787-0 28342636

[5] Koch PJ Walsh MJ Schmelz M Goldschmidt MD Zimbelmann R Franke WW. Identification of desmoglein, a constitutive desmosomal glycoprotein, as a member of the cadherin family of cell adhesion molecules. Eur J Cell Biol. (1990) 53:1–12. 1706270

[6] Amagai M Klaus-Kovtun V Stanley JR. Autoantibodies against a novel epithelial cadherin in pemphigus vulgaris, a disease of cell adhesion. Cell (1991) 67:869–77. 1720352 10.1016/0092-8674(91)90360-b

[7] Wheeler GN Parker AE Thomas CL Ataliotis P Poynter D Arnemann J. Desmosomal glycoprotein DGI, a component of intercellular desmosome junctions, is related to the cadherin family of cell adhesion molecules. Proc Natl Acad Sci USA. (1991) 88:4796–800. 1711210 10.1073/pnas.88.11.4796 PMC51753

[8] Ishii K Amagai M Hall RP Hashimoto T Takayanagi A Gamou S. Characterization of autoantibodies in pemphigus using antigen-specific enzyme-linked immunosorbent assays with baculovirus-expressed recombinant desmogleins. J Immunol. (1997) 159:2010–7. 9257868

[9] Eyre RW Stanley JR. Identification of pemphigus vulgaris antigen extracted from normal human epidermis and comparison with pemphigus foliaceus antigen. J Clin Invest. (1988) 81:807–12. 10.1172/JCI113387 3343340 PMC442529

[10] Ding X Aoki V Mascaro JM Jr Lopez-Swiderski A Diaz LA Fairley JA. Mucosal and mucocutaneous (generalized) pemphigus vulgaris show distinct autoantibody profiles. J Invest Dermatol. (1997) 109:592–6. 9326396 10.1111/1523-1747.ep12337524

[11] Amagai M Komai A Hashimoto T Shirakata Y Hashimoto K Yamada T. Usefulness of enzyme-linked immunosorbent assay using recombinant desmogleins 1 and 3 for serodiagnosis of pemphigus. Br J Dermatol. (1999) 140:351–7. 10233237 10.1046/j.1365-2133.1999.02752.x

[12] Mahoney MG Wang Z Rothenberger K Koch PJ Amagai M Stanley JR. Explanations for the clinical and microscopic localization of lesions in pemphigus foliaceus and vulgaris. J Clin Invest. (1999) 103:461–8. 10.1172/JCI5252 10021453 PMC408100

[13] Ahmed AR Carrozzo M Caux F Cirillo N Dmochowski M Alonso AE. Monopathogenic vs multipathogenic explanations of pemphigus pathophysiology. Exp Dermatol. (2016) 25:839–46. 10.1111/exd.13106 27305362

[14] Sajda T Hazelton J Patel M Seiffert-Sinha K Steinman L Robinson W. Multiplexed autoantigen microarrays identify HLA as a key driver of anti-desmoglein and -non-desmoglein reactivities in pemphigus. Proc Natl Acad Sci USA. (2016) 113:1859–64. 10.1073/pnas.1525448113 26831096 PMC4763733

[15] Schmidt E Spindler V Eming R Amagai M Antonicelli F Baines JF. Meeting report of the pathogenesis of pemphigus and pemphigoid meeting in munich, september 2016. J Invest Dermatol. (2017) 137:1199–203. 10.1016/j.jid.2017.01.028 28390814

[16] Amagai M Ahmed AR Kitajima Y Bystryn JC Milner Y Gniadecki R Are desmoglein autoantibodies essential for the immunopathogenesis of pemphigus vulgaris, or just “witnesses of disease”? Exp Dermatol (2006) 15:815–31. 10.1111/j.1600-0625.2006.00499_1.x 16984264

[17] Chernyavsky AI Arredondo J Piser T Karlsson E Grando SA. Differential coupling of M1 muscarinic and alpha7 nicotinic receptors to inhibition of pemphigus acantholysis. J Biol Chem. (2008) 283:3401–8. 10.1074/jbc.M704956200 18073210

[18] Kalantari-Dehaghi M Chen Y Deng W Chernyavsky A Marchenko S Wang PH. Mechanisms of mitochondrial damage in keratinocytes by pemphigus vulgaris antibodies. J Biol Chem. (2013) 288:16916–25. 10.1074/jbc.M113.472100 23599429 PMC3675624

[19] Cipolla GA Park JK Lavker RM Petzl-Erler ML. Crosstalk between signaling pathways in pemphigus: a role for endoplasmic reticulum stress in p38 mitogen-activated protein kinase activation? Front Immunol. (2017) 8:1022. 10.3389/fimmu.2017.01022 28928733 PMC5591886

[20] Spindler V Eming R Schmidt E Amagai M Grando S Jonkman MF. Mechanisms causing loss of keratinocyte cohesion in pemphigus. J Invest Dermatol. 138:32–7. (2017). 10.1016/j.jid.2017.06.022 29037765

[21] Eming R Sticherling M Hofmann SC Hunzelmann N Kern JS Kramer H. S2k guidelines for the treatment of pemphigus vulgaris/foliaceus and bullous pemphigoid. J Dtsch Dermatol Ges. (2015) 13:833–44. 10.1111/ddg.12606 26213827

[22] Hertl M Jedlickova H Karpati S Marinovic B Uzun S Yayli S. Pemphigus. S2 Guideline for diagnosis and treatment–guided by the European Dermatology Forum (EDF) in cooperation with the European Academy of Dermatology and Venereology (EADV). J Eur Acad Dermatol Venereol. (2015) 29:405–14. 10.1111/jdv.12772 25338479

[23] Joly P Maho-Vaillant M Prost-Squarcioni C Hebert V Houivet E Calbo S. First-line rituximab combined with short-term prednisone versus prednisone alone for the treatment of pemphigus (Ritux 3): a prospective, multicentre, parallel-group, open-label randomised trial. Lancet (2017) 389:2031–40. 10.1016/S0140-6736(17)30070-3 28342637

[24] Schmidt E Zillikens D. Immunoadsorption in dermatology. Arch Dermatol Res. (2010) 302:241–53. 10.1007/s00403-009-1024-9 20049466

[25] Waschke J Spindler V. Desmosomes and extradesmosomal adhesive signaling contacts in pemphigus. Med Res Rev. (2014) 34:1127–45. 10.1002/med.21310 24549583

[26] Langenhan J Dworschak J Saschenbrecker S Komorowski L Schlumberger W Stocker W. Specific immunoadsorption of pathogenic autoantibodies in pemphigus requires the entire ectodomains of desmogleins. Exp Dermatol. (2014) 23:253–9. 10.1111/exd.12355 24533885

[27] Schmidt E Klinker E Opitz A Herzog S Sitaru C Goebeler M. Protein A immunoadsorption: a novel and effective adjuvant treatment of severe pemphigus. Br J Dermatol. (2003) 148:1222–9. 10.1046/j.13652133.2003.05302.x 12828752

[28] Kasperkiewicz M Shimanovich I Meier M Schumacher N Westermann L Kramer J. Treatment of severe pemphigus with a combination of immunoadsorption, rituximab, pulsed dexamethasone and azathioprine/mycophenolate mofetil: a pilot study of 23 patients. Br J Dermatol. (2012) 166:154–60. 10.1111/j.1365-2133.2011.10585.x 21910700

[29] Groth S Recke A Vafia K Ludwig RJ Hashimoto T Zillikens D. Development of a simple enzyme-linked immunosorbent assay for the detection of autoantibodies in anti-p200 pemphigoid. Br J Dermatol. (2011) 164:76–82. 10.1111/j.1365-2133.2010.10056.x 20854435

[30] Schmidt E Gutberlet J Siegmund D Berg D Wajant H Waschke J Apoptosis is not required for acantholysis in pemphigus vulgaris. Am J Physiol Cell Physiol. (2009) 296:C162–72. 10.1152/ajpcell.00161.2008 18987254

[31] Spindler V Rotzer V Dehner C Kempf B Gliem M Radeva M. Peptide-mediated desmoglein 3 crosslinking prevents pemphigus vulgaris autoantibody-induced skin blistering. J Clin Invest. (2013) 123:800–11. 10.1172/JCI60139 23298835 PMC3561799

[32] Payne AS Ishii K Kacir S Lin C Li H Hanakawa Y. Genetic and functional characterization of human pemphigus vulgaris monoclonal autoantibodies isolated by phage display. J Clin Invest. (2005) 115:888–99. 10.1172/JCI24185 15841178 PMC1070425

[33] Anhalt GJ Labib RS Voorhees JJ Beals TF Diaz LA. Induction of pemphigus in neonatal mice by passive transfer of IgG from patients with the disease. N Engl J Med. (1982) 306:1189–96. 10.1056/NEJM198205203062001 7040962

[34] Dworschak J Recke A Freitag M Ludwig RJ Langenhan J Kreuzer OJ. Mapping of B cell epitopes on desmoglein 3 in pemphigus vulgaris patients by the use of overlapping peptides. J Dermatol Sci. (2012) 65:102–9. 10.1016/j.jdermsci.2011.11.012 22261006

[35] Amagai M Matsuyoshi N Wang ZH Andl C Stanley JR. Toxin in bullous impetigo and staphylococcal scalded-skin syndrome targets desmoglein 1. Nat Med. (2000) 6:1275–7. 10.1038/81385 11062541

[36] Meyersburg D Schmidt E Kasperkiewicz M Zillikens D. Immunoadsorption in dermatology. Ther Apher Dial. (2012) 16:311–20. 10.1111/j.1744-9987.2012.01075.x 22817118

[37] Amagai M Hashimoto T Shimizu N Nishikawa T. Absorption of pathogenic autoantibodies by the extracellular domain of pemphigus vulgaris antigen (Dsg3) produced by baculovirus. J Clin Invest. (1994) 94:59–67. 10.1172/JCI117349 8040292 PMC296282

[38] Grando SA. Pemphigus autoimmunity: hypotheses and realities. Autoimmunity (2012) 45:7–35. 10.3109/08916934.2011.606444 21939410 PMC3251002

[39] Mao X Choi EJ Payne AS. Disruption of desmosome assembly by monovalent human pemphigus vulgaris monoclonal antibodies. J Invest Dermatol. (2009) 129:908–18. 10.1038/jid.2008.339 19037235 PMC2743719

[40] Jennings JM Tucker DK Kottke MD Saito M Delva E Hanakawa Y. Desmosome disassembly in response to pemphigus vulgaris IgG occurs in distinct phases and can be reversed by expression of exogenous Dsg3. J Invest Dermatol. (2011) 131:706–18. 10.1038/jid.2010.389 21160493 PMC3235416

[41] Tsunoda K Ota T Aoki M Yamada T Nagai T Nakagawa T. Induction of pemphigus phenotype by a mouse monoclonal antibody against the amino-terminal adhesive interface of desmoglein 3. J Immunol. (2003) 170:2170–8. 10.4049/jimmunol.170.4.2170 12574390

[42] Schulze K Galichet A Sayar BS Scothern A Howald D Zymann H. An adult passive transfer mouse model to study desmoglein 3 signaling in pemphigus vulgaris. J Invest Dermatol. (2012) 132:346–55. 10.1038/jid.2011.299 21956125 PMC3258361

[43] Nguyen VT Ndoye A Grando SA. Pemphigus vulgaris antibody identifies pemphaxin. a novel keratinocyte annexin-like molecule binding acetylcholine. J Biol Chem. (2000) 275:29466–76. 10.1074/jbc.M003174200 10899159

[44] Nguyen VT Ndoye A Grando SA. Novel human alpha9 acetylcholine receptor regulating keratinocyte adhesion is targeted by Pemphigus vulgaris autoimmunity. Am J Pathol. (2000) 157:1377–91. 10.1016/S0002-9440(10)64651-2 11021840 PMC1850172

[45] Spindler V Heupel WM Efthymiadis A Schmidt E Eming R Rankl C. Desmocollin 3-mediated binding is crucial for keratinocyte cohesion and is impaired in pemphigus. J Biol Chem. (2009) 284:30556–64. 10.1074/jbc.M109.024810 19717567 PMC2781610

[46] Mao X Nagler AR Farber SA Choi EJ Jackson LH Leiferman KM. Autoimmunity to desmocollin 3 in pemphigus vulgaris. Am J Pathol. (2010) 177:2724–30. 10.2353/ajpath.2010.100483 20952584 PMC2993297

[47] Rafei D Muller R Ishii N Llamazares M Hashimoto T Hertl M. IgG autoantibodies against desmocollin 3 in pemphigus sera induce loss of keratinocyte adhesion. Am J Pathol. (2011) 178:718–23. 10.1016/j.ajpath.2010.10.016 21281804 PMC3069870

[48] Chen J Den Z Koch PJ. Loss of desmocollin 3 in mice leads to epidermal blistering. J Cell Sci. (2008) 121(Pt. 17):2844–9. 10.1242/jcs.031518 18682494 PMC2659849

[49] Muller R Heber B Hashimoto T Messer G Mullegger R Niedermeier A. Autoantibodies against desmocollins in European patients with pemphigus. Clin Exp Dermatol. (2009) 34:898–903. 10.1111/j.1365-2230.2009.03241.x 19456767

[50] Brandt O Rafei D Podstawa E Niedermeier A Jonkman MF Terra JB. Differential IgG recognition of desmoglein 3 by paraneoplastic pemphigus and pemphigus vulgaris sera. J Invest Dermatol. (2012) 132:1738–41. 10.1038/jid.2012.1 22318391

[51] Ishii N Teye K Fukuda S Uehara R Hachiya T Koga H. Anti-desmocollin autoantibodies in nonclassical pemphigus. Br J Dermatol. (2015) 173:59–68. 10.1111/bjd.13711 25640111

[52] Mindorf S Dettmann IM Kruger S Fuhrmann T Rentzsch K Karl I. Routine detection of serum antidesmocollin autoantibodies is only useful in patients with atypical pemphigus. Exp Dermatol. (2017) 26:1267–70. 10.1111/exd.13409 28815795

